# Inhibition of SARS-CoV-2 wild-type (Wuhan-Hu-1) and Delta (B.1.617.2) strains by marine sulfated glycans

**DOI:** 10.1093/glycob/cwac042

**Published:** 2022-07-05

**Authors:** Rohini Dwivedi, Poonam Sharma, Marwa Farrag, Seon Beom Kim, Lauren A Fassero, Ritesh Tandon, Vitor H Pomin

**Affiliations:** Department of BioMolecular Sciences, University of Mississippi, Oxford, MS 38677, USA; Department of Microbiology and Immunology, University of Mississippi Medical Center, Jackson, MS 39216, USA; Department of BioMolecular Sciences, University of Mississippi, Oxford, MS 38677, USA; Department of Pharmacognosy, Faculty of Pharmacy, Assiut University, Assiut 71515, Egypt; Department of BioMolecular Sciences, University of Mississippi, Oxford, MS 38677, USA; Department of Microbiology and Immunology, University of Mississippi Medical Center, Jackson, MS 39216, USA; Department of BioMolecular Sciences, University of Mississippi, Oxford, MS 38677, USA; Department of Microbiology and Immunology, University of Mississippi Medical Center, Jackson, MS 39216, USA; Department of Medicine, University of Mississippi Medical Center, Jackson, MS 39216, USA; Department of BioMolecular Sciences, University of Mississippi, Oxford, MS 38677, USA; Research Institute of Pharmaceutical Sciences, School of Pharmacy, University of Mississippi, Oxford, Mississippi, 38677, USA

**Keywords:** fucosylated chondroitin sulfate, nuclear magnetic resonance, SARS-CoV-2, sulfated fucan, viral inhibition

## Abstract

The Coronavirus disease pandemic has steered the global therapeutic research efforts toward the discovery of potential anti-severe acute respiratory syndrome coronavirus (SARS-CoV-2) molecules. The role of the viral spike glycoprotein (S-protein) has been clearly established in SARS-CoV-2 infection through its capacity to bind to the host cell surface heparan sulfate proteoglycan (HSPG) and angiotensin-converting enzyme-2. The antiviral strategies targeting these 2 virus receptors are currently under intense investigation. However, the rapid evolution of the SARS-CoV-2 genome has resulted in numerous mutations in the S-protein posing a significant challenge for the design of S-protein-targeted inhibitors. As an example, the 2 key mutations in the S-protein receptor-binding domain (RBD), L452R, and T478K in the SARS-CoV-2 Delta variant (B.1.617.2) confer tighter binding to the host epithelial cells. Marine sulfated glycans (MSGs) demonstrate excellent inhibitory activity against SARS-CoV-2 via competitive disruption of the S-protein RBD-HSPG interactions and thus have the potential to be developed into effective prophylactic and therapeutic molecules. In this study, 7 different MSGs were evaluated for their anti-SARS-CoV-2 activity in a virus entry assay utilizing a SARS-CoV-2 pseudovirus coated with S-protein of the wild-type (Wuhan-Hu-1) or the Delta (B.1.617.2) strain. Although all tested MSGs showed strong inhibitory activity against both strains, no correlations between MSG structural features and virus inhibition could be drawn. Nevertheless, the current study provides evidence for the maintenance of inhibitory activity of MSGs against evolving SARS-CoV-2 strains.

## Introduction

Coronavirus disease (COVID-19) has been responsible for over 6.3 million deaths globally in a short span of 2 years (World Health Organization [WHO] Covid Dashboard). The causal pathogen of this pandemic is the severe acute respiratory syndrome coronavirus (SARS-CoV-2), an enveloped betacoronavirus (Hu et al. 2020). This virus expresses a heavily glycosylated spike protein (S-protein) on its envelope (Belouzard et al. 2012). The vaccines have been able to prevent new infections and restrict the severe outcomes of COVID-19; however, the chances of reinfections in vaccinated people cannot be eliminated, especially with the rapid emergence of variants of concerns (VOCs) of SARS-CoV-2 with increased transmission rates and the likelihood of evading host immune responses.

The S-protein expressed on the envelope of SARS-CoV-2 is the primary mode of contact with the host cell heparan sulfate proteoglycans (HSPGs), thus determining viral tropism, entry, and pathogenesis (Belouzard et al. 2012). The S-protein is a transmembrane glycoprotein which possesses 2 subunits. The S1 subunit, which is capable of interacting with the angiotensin-converting enzyme-2 (ACE2) receptor and the S2 subunit, which helps in membrane fusion, an event necessary for the internalization of the virus particle in the host cell (Belouzard et al. 2012; Tian et al. 2021).

Natural evolution of viruses involves multiple genetic mutations. However, only those capable of changing the important protein structure of the virus lead to consequences in terms of virulence, transmissibility, infectivity, and severity of the disease (Callaway 2020). SARS-CoV-2 wild-type (WT) (Wuhan-Hu-1) strain has acquired many crucial mutations over a short period of time, giving rise to various VOCs (Tian et al. 2021). Delta variant (B.1.617.2), first identified in India in October 2020 (WHO database), has so far been the most aggressive variant (Saito et al. 2021). The high pathogenicity of delta variant is linked to the 9 amino acid mutations in its S-protein: T19R, G142D, FR156-157del, R158G, L452R, T478K, D614G, P681R, and D950N (Tian et al. 2021). The L452R, T478K, and P681R are key mutations because they result in significant immune evasion and high transmissibility (Planas et al. 2021; Tian et al. 2021). The L452R mutation is present in the receptor-binding domain (RBD), a region of the S-protein involved in binding to the ACE2 receptor. This mutation aids in the stabilization of the S-protein-ACE2 interaction, therefore increasing the molecular binding affinity and viral infectivity (Tian et al. 2021). Substitution of the threonine with lysine in T478K changes the electrostatic surface charge of the viral RBD, strengthening the ACE2 binding (Goher et al. 2021). The mutation P681R has been shown to increase proteolytic cleavage caused by furin between the S1 and S2 subunits of the SARS-CoV-2 S-protein (Tian et al. 2021), thus increasing the rate of viral fusion and increasing the spread of infection (Saito et al. 2021). The L452R mutation has been shown to significantly contribute to the immune escaping ability of the virus when examined against vaccine-elicited monoclonal antibodies and convalescent plasma (Tian et al. 2021).

Development of therapeutic strategies capable of inhibiting the infectivity of a broad range of emerging SARS-CoV-2 VOCs in a reasonably consistent manner is critical. As S-protein binding to the host cell surface HSPGs is one of the first and critical steps in the establishment of the infection, abrogation of the same molecular complex would be able to reduce the infectivity of SARS-CoV-2 (Clausen et al. 2020). The competitive inhibition of S-protein-HSPG binding by administering sulfated glycans like glycosaminoglycans (GAGs) or GAG mimetics, e.g. marine sulfated glycans (MSGs) by our group and others, has shown a significant decrease in the SARS-CoV-2 infectivity (Wuhan-Hu-1 strain)(Jin et al. 2020; Kim et al. 2020; Mycroft-West et al. 2020; Dwivedi et al. 2021; Song et al. 2021; Tandon et al. 2021; Guimond et al. 2022; Kim et al. 2022). In our previous studies with Wuhan-Hu-1 strain S-protein RBD, significant inhibition was achieved by MSGs (Dwivedi et al. 2021). Therefore, it is logical to start testing the MSGs against emerging VOCs. In the current study, we investigate the anti-SARS-CoV-2 activity of MSGs comparatively against both the SARS-CoV-2 WT (Wuhan-Hu-1) and Delta (B.1.617.2) strains to examine if the acquired mutations in the Delta variant would lead to any change in the anti-SARS-CoV-2 activity of the MSGs.

## Results and discussion

### Structural features of MSGs

**Fig 1:**
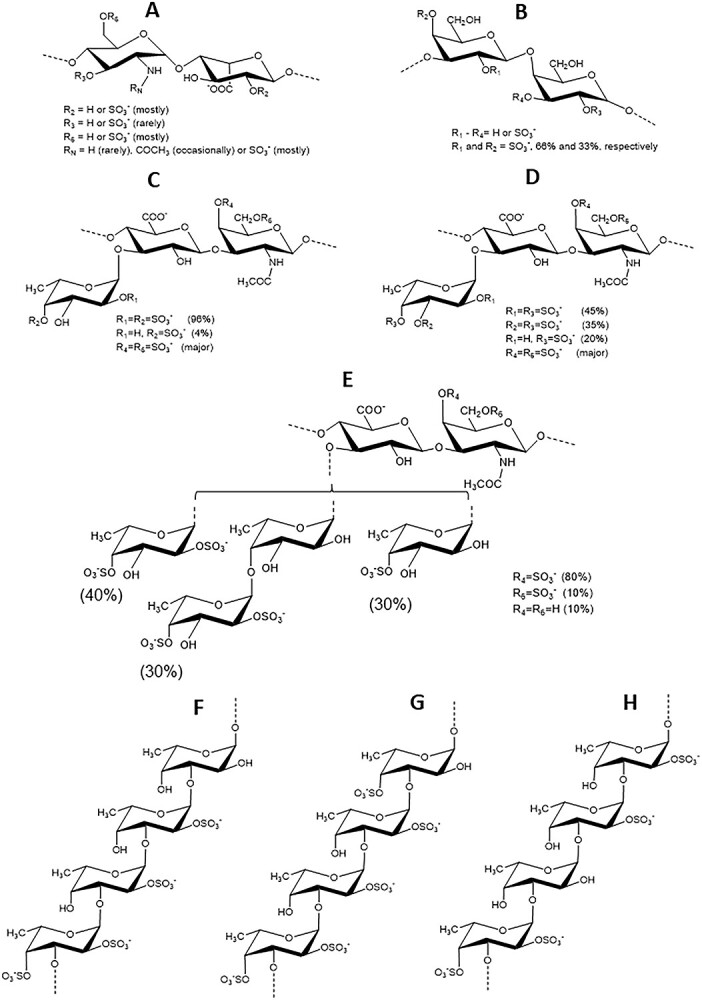
Structural representations of sulfated glycans assayed for anti-SARS-CoV-2 activity. A) Heparin, B) BoSG, C) IbFucCS, D) HfFucCS, E) PpFucCS, F) IbSF, G) LvSF, and H) HfSF.

Figure 1 displays all sulfated glycans screened in inhibitory activity against SARS-CoV-2 WT (Wuhan-Hu-1) and Delta (B.1.617.2) strains. The MSGs examined comparatively to heparin, unfractionated heparin (UFH) (Fig. 1A), include 3 fucosylated chondroitin sulfates (FucCSs) from sea cucumbers, 3 sulfated fucans (SFs) from 2 sea cucumbers and 1 sea urchin, and 1 sulfated galactan (SG) from a red alga. The species and structures of the FucCS series are *Isostichopus badionotus* (IbFucCS) {→3)-β-GalNAc4,6S-(1→4)-β-GlcA-[(3→1)Y]-(1→}_*n*_, where Y = α-Fuc2,4S (96%), or α-Fuc4S (4%) (Fig. 1C), *Holothuria floridana* (HfFucCS) {→3)-β-GalNAc4,6S-(1→4)-β-GlcA-[(3→1)Y]-(1→}*_n_*, where Y = αFuc2,4S (45%), α-Fuc3,4S (35%), or α-Fuc4S (20%) (Fig. 1D) and *Pentacta pygmaea* (PpFucCS) {→3)-β-GalNAcX-(1→4)-β-GlcA-[(3→1)Y]-(1→}*_n_*, where X = 4S (80%), 6S (10%) or nonsulfated (10%), Y = α-Fuc2,4S (40%), α-Fuc2,4S-(1→4)-α-Fuc (30%), or α-Fuc4S (30%) (Fig. 1E). The other glycans are the SG from *Botryocladia occidentalis* (BoSG) [3-β-Gal2R_1_4R_2_-(1→4)-α-Gal2R3R-(1→]*_n_* in which R = SO_3_^−^ or OH, R_1_ and R_2_ = 66% and 33% sulfation, respectively (Fig. 1B), the SF from the sea urchin *Lytechinus variegatus* (LvSF) (Fig. 1G), [→3)-α-Fuc2,4S-(1→3)-α-Fuc2S-(1→3)-α-Fuc2S-(1→3)-α-Fuc4S-(1→]*_n_*, the SF from the sea cucumber *H. floridana* (HfSF) (Fig. 1H), [→3)-α-Fuc2,4S-(1→3)-α-Fuc-(1→3)-α-Fuc2S-(1→3)-α-Fuc2S-(1→]_*n*,_ and from *I. badionotus* (IbSF) (Fig. 1F), [→3)-α-Fuc2,4S-(1→3)-α-Fuc2S-(1→3)-α-Fuc2S-(1→3)-α-Fuc-(1→]*_n_*.

### Structural integrity and molecular weights

**Fig 2:**
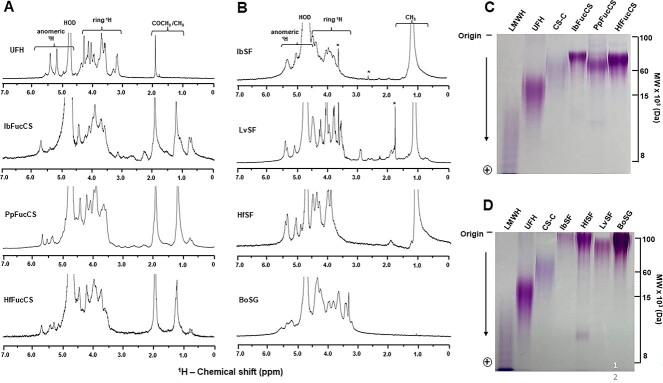
A and B) 1D ^1^H NMR spectra and B and C) PAGE of UFH and MSGs. A) ^1^H NMR spectra (δ_H_ expansion, 7.0–0.0 ppm) of A) GAGs and B) homopolysaccharides acquired in D_2_O at 25 °C on 400-MHz Bruker NMR instrument. The spectra of each sulfated glycan sample are indicated accordingly in the panel. The NMR signals corresponding to anomeric and ring protons of polysaccharides have been labeled on top of spectral stacks. The signals indicated with asterisks are from solvent. MW distribution of MSGs, C) IbFucCS, PpFucCS, HfFucCS and D) LvSF, IbSF, HfSF, and BosG, analyzed on a 22% PAGE using the GAGs of known MWs as markers: low MW heparin (LMWH, of ~8 kDa); UFH of ~15 kDa, and chondroitin sulfate C-type (CS-C of ~60 kDa).

All sulfated glycans studied here are natural products isolated from digestions of different biological sources. The structural integrity and reliable purity levels of all these sulfated glycans was confirmed by 1D ^1^H nuclear magnetic resonance (NMR) spectroscopy (Fig. 2A and B), as they show typical spectral profiles as reported previously (Tandon et al. 2021 for UFH, Farias et al. 2000 for BoSG, Chen et al. 2011 for IbFucCS, Dwivedi et al. 2021 for PpFucCS, Shi et al. 2019 for HfFucCS, Chen et al. 2012 for IbSF, Pomin et al. 2005 for LvSF, and Shi et al. 2019 for HfSF). The purified MSGs were further analyzed by 22% polyacrylamide gel electrophoresis (PAGE) (Fig. 2C and D) to understand polydispersity distribution and to correlate the different molecular weights (MWs) of MSGs with their anti-SARS-CoV-2 effects. Their MW distribution was assessed in comparison with mammalian GAGs of known MWs. The series of holothurian FucCS showed bands between ~60 and ~100 kDa (Fig. 2C). The MW distribution of the homopolysaccharides composed of galactose (BoSG) and fucose (IbSF, HfSF, and LvSF) indicates higher MW for these polymers, ≥100 kDa (Fig. 2D). The MWs of the series of FucCS and SF/SG are in accordance with previous publications (Shi et al. 2019; Dwivedi et al. 2021; Zoepfl et al. 2021), confirming therefore the structural integrity.

### Anti-SARS-CoV-2 Wuhan and Delta strains and cytotoxicity of MSGs

**Fig 3:**
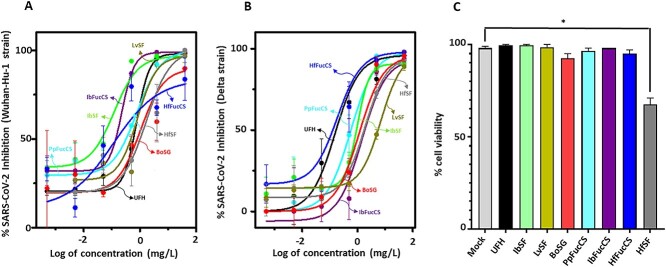
Anti-SARS-CoV-2 and cytotoxic activity of UFH and MSG. UFH and MSGs were assayed for their potential to inhibit GFP transduction in HEK-293T-hACE2 cells infected with pseudotyped SARS-Cov-2 S-proteins of A) Wuhan-Hu-1 and B) Delta (B.1.617.2) strains. Normalized values from the assay were analyzed by the nonlinear regression to fit a dose–response curve using the least-squares method, considering each replicate value as an individual point. The plotted curve shows percentage of SARS-CoV-2 WT and Delta inhibition in a (log) concentration-dependent manner. Curves in the plot represent the following sulfated glycans—UFH (black), algal BoSG (red), sea urchin LvSF (yellow), holothurian SFs-IbSF (green), HfSG (gray), and holothurian FucCSs—IbFucCS (purple), PpFucCS (cyan), and HfFucCS (blue). C) Bar plots represent percentage cell viability of HEK-293T-hACE2 cells when treated with the above mentioned sulfated glycans at the highest examined concentration of 50 mg/L. All sugars were nonsignificant (*P* > 0.05) except HfSF (*P* < 0.05) indicated by *.

MSGs were examined for their ability to inhibit SARS-CoV-2 entry into HEK-293T-hACE2 cells using a baculovirus pseudotyped with S-proteins of WT (Wuhan-Hu-1) (Fig. 3A and Table S1) and Delta (B.1.617.2) (Fig. 3B and Table S2) strains and encoding green fluorescent protein (GFP). The IC_50_ values of the MSGs (Table 1) demonstrated generally similar activity of heparin (IC_50_ = 0.6 mg/L) against the Wuhan strain (range of 0.1–1.0 mg/L) and generally (slightly) weaker activity than heparin (IC_50_ = 0.2 mg/L) against the Delta strain (range of 0.1–5.9 mg/L). In the cytotoxicity assays (trypan blue exclusion) on HEK-293T-hACE2 cells, all tested MSGs, except HfSF, exhibited no significant cell death even at the highest concentration of 50 mg/L and 60 h of continuous exposure as compared to the mock-treated cells (Fig. 3B and Table S3).

**Table 1:** IC_50_ of anti-SARS-CoV-2 WT (Wuhan-Hu-1) and Delta (B.1.617.2) actions of UFH and MSGs.

Sulfated glycan	IC_50_ (mg/L)^a^ ± SD^b^	
	Wuhan-Hu-1	Delta
UFH	0.6 ± 0.037	0.2 ± 0.030
IbFucCS	0.1 ± 0.005	1.6 ± 0.216
BoSG	0.9 ± 0.262	1.3 ± 0.260
IbSF	0.1 ± 0.005	1.0 ± 0.126
PpFucCS	0.3 ± 0.061	0.6 ± 0.104
HfFucCS	0.1 ± 0.014	0.1 ± 0.010
HfSF	1.0 ± 0.180	1.8 ± 0.324
LvSF	0.5 ± 0.032	5.9 ± 0.620

^a^IC_50_ values of anti-SARS-CoV-2 inhibitory activity of UFH and MSGs were determined against HEK-293T-hACE2 cells infected with baculovirus pseudotyped with SARS-CoV-2 WT (Wuhan-Hu-1) and Delta (B.1.617.2) s-proteins.

^b^Standard deviation (SD) values from triplicates.

### Concluding remarks

In this study, 7 MSGs were examined, comparatively to heparin, regarding their capacities to block 2 strains, WT (Wuhan-Hu-1) and Delta (B.1.617.2), of SARS-CoV-2. Although each sulfated glycan showed its own inhibitory capacity, the inhibitory activities of the MSGs strains were generally similar and (slightly) weaker, respectively, against Wuhan and Delta strains. No correlation between structural features (sulfation pattern, MW, monosaccharide composition, glycosidic linkage, and/or anomeric configuration) and anticoronaviral activity was observed. Nevertheless, the current study provides evidence for the maintenance of inhibitory activity of MSGs against evolving SARS-CoV-2 strains.

## Materials and methods

### Materials

Sea cucumbers, *I. badionotus*, *P. pygmaea* and *H. floridana*, were obtained from the Gulf Specimen Lab (Gulf of Mexico, Florida Keys). Papain, Sephadex G15 medium, and DEAE Sephacel resin were purchased from Sigma (St. Louis, MO, United States). LMWH, UFH (180 IU/mg), and CS-C were purchased from Sigma. Baculovirus pseudotyped with SARS-CoV-2 WT and Delta variant S-protein containing a GFP reporter was obtained from Montana Molecular (Bozeman, MT, United States). HEK-293T-hACE2 cells were obtained from BEI resources. Deuterium oxide (D_2_O) (D 99.90%) was purchased from Cambridge Isotope Laboratories, Inc. (Andover, MA, United States). NMR tubes (3 mm) were purchased from VWR International (Radnor, PA, United States).

### Extraction of MSGs

The sulfated glycans—IbSF, IbFucCS, PpFucCS, HfFucCS, and HfSF were extracted from the body wall of sea cucumbers—*I. badionotus*, *P. pygmaea*, and *H. floridana* following the proteolytic digestion procedure reported earlier (Chen et al. 2011, 2012; Dwivedi et al. 2021)*.* The 100 mg of dried body wall was digested using 0.1 mg of papain (0.5–2 U/mg) at 60 °C (24 h) in 2 mL of digestion buffer (5 mM of cysteine, 5 mM of EDTA in 0.1 M of sodium acetate buffer, pH 6.0). The digested mixture was centrifuged (4,000 rpm for 30 min) and the supernatant was precipitated using 2 volumes of 95% ethanol. The precipitate was kept at −20 °C for 24 h before centrifugation at 4,000 rpm for 30 min. Precipitate was dissolved in water, dialyzed 3 times against distilled water, and lyophilized. BoSG and LvSF were isolated from the body wall of the red alga *B. occidentalis* and the egg jelly of the female gametes of *L. variegatus*, as reported earlier (Pomin et al. 2021).

### Purification of MSGs

The crude extract (50 mg) was fractionated by anion exchange chromatography (packed with DEAE Sepahcel resin) column (2.5 × 20 cm). A linear gradient of NaCl (in 0.1 M NaOAc, pH 6.0) from 0 to 3 M was applied at a flow rate of 18 mL/h. The eluted fractions were assayed for the presence of sulfated glycans by 1,9–dimethylmethylene blue reagent. Fractions were pooled and dialyzed 3 times against water and were lyophilized. Sugars were further purified on a size exclusion column Sephadex G15 (1 × 30 cm).

### NMR analysis

The 1D ^1^H NMR spectra of UFH and MSGs (IbSF, IbFucCS, PpFucCS, HfFucCS, and HfSF) were acquired at 25 °C on 400-MHz Bruker Avance III HD using a 5-mm BBFO RT probe. The NMR sample was prepared in a 3-mm NMR tube by dissolving 3–5 mg of pure polysaccharide in 200 μL D_2_O (99.90%). The 64 NMR scans were acquired for each sample involving a relaxation delay of 1 sec with a total acquisition time of 1 min 41 s. The acquired proton NMR data was processed and analyzed using MestreNova 14.1.0 and TopSpin 4.0 software.

### MW estimation of MSGs

The MW distribution of the purified MSGs was determined on a 1.5-mm thick discontinuous PAGE system having 4% stacking gel and a 22% resolving gel phase. The 10 μg of samples dissolved in loading buffer (50% glycerol, 0.5 M Tris, pH 6.8) were loaded into the wells of the stacking gel. LMWH (~8 kDa), UFH (~15 kDa), and CS-C (~60 kDa) were run alongside the MSGs as MW markers. Electrophoretic migration was carried out at 100 V in 0.25 M of Tris-Glycine running buffer. The migration of bands was tracked by 0.02% bromocresol green dye. Gel was stained using 0.1% (w/v) toluidine blue (in 1% acetic acid) for 1 h and was destained using 1% acetic acid (Vasconcelos et al. 2018; Dwivedi et al. 2021).

### Viral inhibition of MSGs

The virus inhibitor screening was done on human embryonic kidney cells (HEK-293T) expressing human ACE2 (HEK-293T-hACE2 cell line, BEI Resources #NR-52511) plated in a 96-well tissue culture plate using baculovirus pseudotyped with SARS-CoV-2 Wuhan-Hu-1 and Delta S-proteins (#C1110G and #C1123G, respectively) containing a green fluorescent reporter (Montana Molecular, #C1123G) (Dwivedi et al. 2021; Tandon et al. 2021; Yan et al. 2021). Virus titers were confirmed by enumerating GFP-positive transduced cells in a dilution series under a fluorescence microscope (EVOS-FL, Thermo Fisher Scientific) and by multiplying by the dilution factor with the volume plated. Serial dilutions (50, 5, 0.5, 0.05, 0.005, and 0.0005 mg/L) of the test MSGs were made in Dulbecco's modified Eagle's medium in triplicates to the final volume of 100 μL in each well. The controls used were UFH at 50 mg/L and mock-treated cells. The 2.5 μL of the pseudotype virus stock (2 × 10^10^ units per mL) was mixed with the test compounds and was incubated for 1 h, which was then laid over HEK-293T-hACE2 cells plated in a 96-well plate along with 2 mM of sodium butyrate. Plate was then incubated for 60 h and the assay was read on a Cytation 5 automated fluorescence microscope after fixing with 3.7% formaldehyde. The relative IC_50_ values were calculated in Prism 9 (Graphpad Inc.) by plotting normalized values from the assay against the concentrations (log) of MSGs and controls and analyzing by nonlinear regression to fit a dose–response curve using the least-squares method considering each replicate value as an individual point.

### Cytotoxicity of MSGs

Cytotoxic potential of MSGs was examined against HEK-293T-hACE2 cells seeded in 12-welled tissue culture plates following the protocol reported earlier (Dwivedi et al. 2021; Tandon et al. 2021). Briefly, the confluent HEK-293T-hACE2 cells were treated with MSGs at a final concentration of 50 mg/L along with 2 mM of sodium butyrate. The cell viability was estimated via trypan blue exclusion assay upon harvesting the treated cells after 60 h of incubation. Assay readout was measured on a TC20 automated cell counter (BioRad) according to the manufacturer’s protocol.

## Funding

This work was supported by National Institutes of Health (grant numbers 1P20GM130460-01A1-7936 and 1R03NS110996-01A1) and the University of Mississippi to V.H.P. National Aeronautics and Space Administration award #80NSSC19K1603 and NIH award 1R01DE031928-01A1 supported the work in the laboratory of R.T. M.F. is funded by a full scholarship (GM 1110) from the Ministry of Higher Education of the Arab Republic of Egypt.

## Conflict of interest statement

None declared.

## Supplementary Material

supplementary_material-07172022_cwac042
