# Supplementary material for: Inhibition of SARS-CoV-2 wild-type (Wuhan-Hu-1) and Delta (B.1.617.2) strains by marine sulfated glycans
Source: Glycobiology. 2022 Jul 5;32(10):849–54. doi: 10.1093/glycob/cwac042 (PMC9487896; doi:10.1093/glycob/cwac042)
Supplement: supplementary_material-07172022_cwac042 [file supplementary_material-07172022_cwac042.docx]

**Supplementary Material**

**Inhibition of SARS-CoV-2 Wild-Type (Wuhan-Hu-1) and Delta (B.1.617.2) Strains by Marine Sulfated Glycans**

Rohini Dwivedi, Poonam Sharma, Marwa Farrag, Seon Beom Kim, Lauren A. Fassero, Ritesh Tandon, Vitor H. Pomin

| % inhibition of SARS- CoV-2 WT (Wuhan-Hu-1)^a^ | | | | | | | | | | | | | | | | |
| --- | --- | --- | --- | --- | --- | --- | --- | --- | --- | --- | --- | --- | --- | --- | --- | --- |
| **Log conc.** | **UFH** | **Std.Err** | **IbFucCS** | **Std.Err** | **IbSF** | **Std. Err** | **PpFucCS** | **Std.Err** | **BoSG** | **Std.Err** | **LvSF** | **Std.Err** | **HfSF** | **Std.Err** | **HfFucCS** | **Std.Err** |
| **(mg/L)** |  |  |  |  |  |  |  |  |  |  |  |  |  |  |  |  |
|  |  |  |  |  |  |  |  |  |  |  |  |  |  |  |  |  |
| **1.6** | 98.02 | 0.27 | 98.83 | 0.37 | 96.52 | -- | 99.90 | 0.08 | 89.64 | 8.53 | 96.74 | 3.84 | 116.43 | 1.64 | 83.62 | 12.03 |
| **0.6** | 95.29 | 3.47 | 98.79 | 0.48 | 96.71 | 0.39 | 91.79 | 1.08 | 47.63 | 13.43 | 31.29 | 0.17 | 64.62 | 16.22 | 67.63 | 4.39 |
| **-0.3** | 44.26 | 4.44 | 86.82 | 1.10 | 93.81 | 0.27 | 57.45 | -- | 46.59 | 4.86 | 26.21 | 5.15 | 26.44 | 14.19 | 79.50 | 8.10 |
| **-1.3** | 28.91 | 5.01 | 35.95 | 3.45 | 47.18 | 3.57 | 40.65 | 3.79 | 28.70 | 9.13 | 33.41 | 3.09 | 40.83 | 10.17 | 46.21 | 11.10 |
| **-2.3** | 21.75 | 3.42 | 32.06 | 7.33 | 38.41 | 9.45 | 37.61 | 9.77 | -- | -- | -- | -- | 19.87 | 7.65 | 11.12 | 5.20 |
| **-3.3** | 20.54 | 2.41 | 31.95 | 8.26 | 33.85 | -- | 29.56 | 3.75 | 22.11 | 32.52 | -- | -- | 33.52 | 4.32 | 32.94 | 6.54 |

**Table SI.** Percentage of inhibition against SARS-CoV-2 WT (Wuhan-Hu-1) and Delta (B.1.617.2) by UFH and MSGs as measured by HEK-293T-hACE2 cells infected with baculovirus pseudotyped with SARS CoV-2 WT and Delta strains at different glycan concentrations.

| % inhibition of SARS- CoV-2 Delta^a^ | | | | | | | | | | | | | | | | |
| --- | --- | --- | --- | --- | --- | --- | --- | --- | --- | --- | --- | --- | --- | --- | --- | --- |
| **Log conc.** | **UFH** | **Std.Err** | **IbFucCS** | **Std.Err** | **IbSF** | **Std. Err** | **PpFucCS** | **Std.Err** | **BoSG** | **Std.Err** | **LvSF** | **Std.Err** | **HfSF** | **Std.Err** | **HfFucCS** | **Std.Err** |
| **(mg/L)** |  |  |  |  |  |  |  |  |  |  |  |  |  |  |  |  |
|  |  |  |  |  |  |  |  |  |  |  |  |  |  |  |  |  |
| **1.6** | 95.82 | 1.10 | 93.77 | 1.27 | 90.62 | 1.04 | 98.06 | 0.22 | 95.63 | 3.40 | 89.02 | 2.49 | 92.59 | 0.55 | 97.93 | 0.72 |
| **0.6** | 81.26 | 3.25 | 85.87 | 2.13 | 87.77 | 1.93 | 95.39 | 0.66 | 90.30 | 4.96 | 47.17 | 3.00 | 72.82 | 3.39 | 92.93 | 1.14 |
| **-0.3** | 66.95 | 9.96 | 7.78 | 12.93 | 30.08 | 6.40 | 46.81 | 12.53 | 23.83 | 8.66 | 17.03 | 4.34 | 24.92 | 1.21 | 64.21 | 15.45 |
| **-1.3** | 29.69 | 14.81 | 2.89 | 9.37 | 14.59 | 1.34 | 13.19 | 3.48 | 8.76 | 11.67 | 15.18 | 1.15 | 7.62 | 10.70 | -- | 3.42 |
| **-2.3** | 9.30 | 17.33 | 0 | -- | 20.87 | 11.37 | 5.43 | 27.98 | 0.71 | 15.46 | 13.03 | 3.31 | 14.35 | 13.69 | -- | 3.53 |
| **-3.3** | 0 | -- | 0 | -- | 10.78 | 10.19 | 0 | -- | 0 | -- | 8.29 | 5.83 | 7.06 | 9.95 | 16.75 | 11.82 |

^a^Values in the table represent % SARS-CoV-2 activity. Std.Err stands for standard error of mean and was determined by triplicated measurements.

**Table SII.** Cytotoxic potential of UFH and MSGs as examined against HEK-293T-hACE2 cells by trypan blue exclusion assay.

| Control/glycan | % live cells ± Sd^a^ |
| --- | --- |
| Mock | 98 ± 1.41 |
| UFH | 99.5 ± 0.70 |
| IbFucCS | 98 ± 0 |
| BoSG | 92.5 ± 3.53 |
| IbSF | 99.5 ± 0.70 |
| PpFucCS | 96.5 ± 2.12 |
| HfFucCS | 95 ± 2.82 |
| HfSF | 67.5 ± 4.94 |
| LvSF | 98.5 ± 2.12 |

^a^Values represent % cell viability of HEK-293T-hACE2 cells upon treatment with MSGs at a highest concentration of 50 mg/L. Sd represents standard deviation obtained upon duplicate measurements.
